# *Terricella alpina* gen. nov., sp. nov., a Cellulolytic Planctomycete Representing the First Described Soil-Inhabiting Member of the Class *Phycisphaerae*

**DOI:** 10.3390/life16081222

**Published:** 2026-07-23

**Authors:** Anastasia A. Ivanova, Irina S. Kulichevskaya, Daniil G. Naumoff, Gennady S. Kachmazov, Natalia E. Suzina, Svetlana N. Dedysh

**Affiliations:** 1 Winogradsky Institute of Microbiology, Research Center of Biotechnology of the Russian Academy of Sciences, Moscow 119071, Russia; kulich2@mail.ru (I.S.K.); daniil_naumoff@yahoo.com (D.G.N.); dedysh@mail.ru (S.N.D.); 2 State Budgetary Educational Institution, Republican Physics and Mathematics Lyceum-Boarding School Named After M.K. Abaev, Republic of North Ossetia—Alania, Vladikavkaz 362021, Russia; kgssogutfi@yandex.ru; 3 G.K. Skryabin Institute of Biochemistry and Physiology of Microorganisms, Pushchino Scientific Center for Biological Research of the Russian Academy of Sciences, Pushchino 142290, Russia; suzina_nataliya@rambler.ru

**Keywords:** soil planctomycetes, order *Tepidisphaerales*, *Terricella* gen. nov cellulose and xylan degradation, GH5 family, GH10 family

## Abstract

A novel planctomycete, strain T20PH1^T^, was isolated from alpine meadow soil collected at an altitude of 1800 m in the North Caucasus Mountains, Russia. Phylogenomic analysis placed it within the family *Tepidisphaeraceae* of the class *Phycisphaerae*. The closest relatives based on 16S rRNA gene sequence similarity are *Fontivita pretiosa* B-254^T^ (88.9%), ‘*Humisphaera borealis*’ M1803^T^ (88.6%), and *Tepidisphaera mucosa* 2842^T^ (88.4%). Strain T20PH1^T^ is the first soil-derived representative of the class *Phycisphaerae*. Cells of strain T20PH1^T^ are motile, pink-pigmented cocci reproducing by binary fission. This planctomycete is an obligately aerobic chemoorganotroph with growth optima at 25–30 °C and pH 6.0–7.0. A notable functional trait of strain T20PH1^T^ is its ability to grow on cellulose, including microcrystalline, fibrous and carboxymethyl cellulose, as well as on xylan, starch, lichenan and xanthan. The genome comprised a 5.15 Mb chromosome and a 125 kb plasmid with G + C contents of 65.97 and 66.75%, respectively. Genome analysis identified a GH5_5 subfamily cellulase as the most likely enzyme responsible for cellulose degradation by this planctomycete. Based on phenotypic and phylogenomic evidence, strain T20PH1^T^ (=LMG 34157^T^ = KCTC 102499^T^) was classified as representing a novel genus and species, *Terricella alpina* gen. nov., sp. nov.

## 1. Introduction

Members of the phylum *Planctomycetota* are widespread and, in many ways, fascinating bacteria [1,2]. Representatives of this bacterial phylum have been detected in marine and freshwater habitats, wetlands, soils, thermal environments, and host-associated microbiomes, where they play important roles in global carbon and nitrogen cycling [3,4,5,6,7,8,9,10]. The unique features for which planctomycetes are known include the absence of many canonical divisome proteins, including FtsZ, large genomes (up to 12.5 Mb) with nearly half of the encoded proteins lacking functional annotation, numerous membrane invaginations, and a Gram-negative cell envelope, although not entirely typical in its organization [11,12,13,14,15,16,17]. During the last two decades, cultivation efforts and molecular approaches have considerably expanded our understanding of planctomycetal diversity; nevertheless, the number of cultivated representatives remains low. At present, the phylum is subdivided into the classes *Planctomycetia* [18], *Phycisphaerae* [19] and *Candidatus* Brocadiae [20]. Cultivated representatives are unevenly distributed between the first two classes. While nearly 70 genera and more than 100 species have been described within the class *Planctomycetia*, the class *Phycisphaerae* remains comparatively poorly explored, with only 13 genera and 14 species described to date.

The class *Phycisphaerae* currently comprises the orders *Phycisphaerales* [19], *Tepidisphaerales* [21] and *Sedimentisphaerales* [22]. Among them, *Tepidisphaerales* is the least diverse lineage, encompassing only three described genera and species. The first described representative of this order, *Tepidisphaera mucosa* 2842^T^, is a moderately thermophilic facultatively anaerobic bacterium recovered from a terrestrial hot spring in the Baikal Lake region, Russia. This organism is capable of degrading a range of polysaccharides and possesses the smallest genome currently known within the order (3.43 Mb) [21]. Several years later, a mesophilic member of the same lineage, ‘*Humisphaera borealis*’ M1803^T^, was obtained from the upper oxic layer of a eutrophic humic lake in the Russian middle taiga. Similar to *T. mucosa*, strain M1803^T^ utilizes various polymeric substrates but possesses the largest genome among members of *Tepidisphaerales* (7.19 Mb). A particularly intriguing feature of this microorganism is the presence of bacterial microcompartments associated with L-sugar metabolism and likely involved in the processing of metabolic intermediates [23]. The third described member of the order, *Fontivita pretiosa* B-254^T^, originated from water and sediments of a hot spring near Goryachinsk in the Baikal Lake region. Like *T. mucosa*, this species is a moderately thermophilic facultatively anaerobic bacterium, but differs from the other representatives in colony pigmentation and substrate utilization patterns [24]. The substantial physiological and ecological differences among the three described species suggest that *Tepidisphaerales* is more diverse than currently recognized. Consistent with this assumption, cultivation-independent studies indicate that members of this order are widespread in nature. *Tepidisphaerales*-affiliated sequences have been detected in soils, peatlands, freshwater habitats, hot springs and other environments [24]. In particular, previous analyses of environmental 16S rRNA gene datasets demonstrated that the WD2101 lineage, to which *‘H. borealis*’ belongs, is especially abundant in terrestrial ecosystems [23].

While isolating xylan-degrading microorganisms from alpine meadow soil collected at an altitude of 1800 m in the North Caucasus Mountains, we noticed several small pink colonies composed of spherical cells resembling members of the class *Phycisphaerae*. One of these isolates was subsequently confirmed as a pure culture and designated strain T20PH1^T^. Preliminary phylogenetic analyses placed the isolate within the order *Tepidisphaerales*. Since strain T20PH1^T^ was only the fourth cultivated member of this order and the first recovered from soil, the present study was initiated in order to examine physiological and genomic characteristics of strain T20PH1^T^, its ability to degrade plant-derived polysaccharides, such as cellulose and xylan, and to establish a taxonomic position of this novel member of the poorly characterized planctomycetal order *Tepidisphaerales*.

## 2. Materials and Methods

### 2.1. Sampling Site and Isolation Approach

Strain T20PH1^T^ was isolated from alpine meadow soil (pH 6.8) collected at an altitude of 1800 m in the mountainous region of the Republic of North Ossetia-Alania, southern part of the North Caucasus, Russia (42.916680, 43.530460). The vegetation at the sampling site consisted of perennial meadow herbs and grasses, including *Potentilla crantzii*, *Geranium gymnocaulos*, *Gentiana pneumonanthe*, *Aster alpinus* and *Festuca varia*. Soil was collected from a depth of 5–10 cm in August 2023 and transported to the laboratory.

The sample was used to establish an enrichment culture targeting microorganisms involved in the degradation of xylan, a major plant biopolymer in soil ecosystems. For this purpose, 2 g of soil were inoculated into 160 mL glass flasks containing 0.1% birchwood xylan (Fluka) and 30 mL of a soil extract prepared from alpine soil. Enrichment cultures were incubated at room temperature (20–22 °C) for 4 weeks. Isolation was performed by spreading 20 µL aliquots onto medium M31 solidified with 10 g L^−1^ Phytagel (Sigma-Aldrich, Steinheim, Germany). The medium contained (per liter distilled water): 0.1 g KH_2_PO_4_, 20 mL Hutner’s basal salts, 1.0 g N-acetylglucosamine, 0.1 g peptone, 0.1 g yeast extract, and 0.1% xylan, adjusted to pH 6.8 [25]. Plates were incubated at 22 °C for 4 weeks. Small (≤1 mm), pink colonies that developed were selected, examined microscopically, and purified through repeated cultivation. The resulting pure culture, designated strain T20PH1^T^, was maintained on medium M31 and subcultured at 2-month intervals.

### 2.2. Phenotypic Characteristics and Growth Parameters of Strain T20PH1^T^

Physiological characterization of strain T20PH1^T^ was performed in liquid medium M31. Growth was monitored nephelometrically at 600 nm using an Eppendorf BioPhotometer. Temperature tolerance was examined at 4–37 °C, pH tolerance at pH 4.0–8.0. The pH range 5.5–6.0 was established using MES buffer, whereas MOPS buffer was used for pH 6.5–8.0 according to the manufacturer’s instructions. Media with pH values between 4.0 and 5.0 were adjusted with 25% HCl.

Carbon source utilization was tested in a modified mineral version of medium M31 [25] in which N-acetylglucosamine and peptone were omitted and the yeast extract concentration was reduced to 0.005% (*w*/*v*). Individual substrates were supplied at a final concentration of 0.05% (*w*/*v*). Cultures were incubated in 100 mL flasks containing 20 mL medium at room temperature on a rotary shaker for 2 weeks. Control incubations lacking an added carbon source were performed in parallel.

The ability of strain T20PH1^T^ to utilize cellulose was assessed in modified mineral medium M31 supplemented with microcrystalline cellulose, fibrous cellulose, or carboxymethyl cellulose (0.1%, *w*/*v*) as sole carbon sources. Cultures were incubated at room temperature for up to 30 days. Growth was monitored visually and by comparison with control incubations lacking added carbon source.

Growth under anaerobic conditions was tested in anaerobic jars using AnaeroGen anaerobic atmosphere generation systems (Oxoid). Oxidase activity, enzymatic profiles, nitrate reduction, gelatin hydrolysis and other physiological characteristics were determined using API 20NE test kit (bioMérieux) according to the manufacturer’s instructions. Catalase activity was assessed using the standard method [26].

Susceptibility to antibiotics was examined on solid M31 medium using Oxoid antibiotic discs containing ampicillin (10 μg), gentamicin (10 μg), kanamycin (30 μg), neomycin (10 μg), streptomycin (10 μg), chloramphenicol (30 μg), rifampicin (10 μg), tetracycline (10 μg) and lincomycin (10 μg). Growth inhibition zones were recorded after 2 weeks of incubation at room temperature. Only inhibition zones exceeding 2 mm were considered positive.

### 2.3. Morphological Characterization of Strain T20PH1^T^ by Light and Electron Microscopy

Cell morphology and size measurements were performed using a Zeiss Axioplan 2 microscope with Axiovision 4.2 software (Zeiss, Jena, Germany). Ultrathin cell sections for electron microscopy were prepared as described previously [23]. For negative staining, cells were dried onto grids, treated with 1% (*w*/*v*) phosphotungstic acid, and were examined with a JEM-1400 (JEOL, Tokio, Japan) transmission electron microscope.

Cells grown on fibrous cellulose were examined by scanning electron microscopy. Cell pellets were fixed in 1.5% (*v*/*v*) glutaraldehyde prepared in 0.05 M cacodylate buffer (pH 7.2) for 1 h at 4 °C, washed three times with the same buffer, and post-fixed in 1% (*w*/*v*) osmium tetroxide in 0.05 M cacodylate buffer (pH 7.2) for 3 h at room temperature. Samples were dehydrated through a graded ethanol series (30–100%), infiltrated with tert-butanol (Sigma-Aldrich), and freeze-dried using a JFD-320 freeze-drying device (JEOL, Tokio, Japan). Dried samples were mounted on aluminum stubs with conductive carbon tape, sputter-coated with gold using a JFC-1100 sputter coater (JEOL, Tokio, Japan), and examined with a JSM-6510LV scanning electron microscope (JEOL, Tokio, Japan).

### 2.4. Genome Sequencing, Annotation and Analysis

Genomic DNA for sequencing was extracted using the standard cetyltrimethylammonium bromide (CTAB) and phenol-chloroform method. A portion of the isolated genomic DNA was sequenced on a MinION device (Oxford Nanopore, UK) using an R9.4 flow cell, following the Ligation Sequencing kit 1D protocol according to the manufacturer’s instructions. From the remaining genomic DNA, a library was prepared and sequenced on the Illumina MiSeq platform at Algavitapro (Russia). Hybrid assembly of the Illumina and Nanopore reads was performed using Unicycler software version 0.4.9b [27]. Genome annotation was carried out using the PROKKA [28] and BLASTKoala [29] toolkits. Secondary metabolite gene clusters were identified with antiSMASH 7.0 [30]. The search for proteins of bacterial microcompartments was carried out using the BMC caller program [31]. The annotated chromosome and plasmid sequences of strain T20PH1T have been deposited in GenBank under the accession number JBZEMQ000000000.

### 2.5. Search for Cellulase- and Xylanase-Encoding Genes

The CAZy classification [32] of glycoside hydrolases, which is based on amino acid sequence similarity of catalytic domains, was used in this study. Putative glycoside hydrolases encoded in the genome of strain T20PH1^T^ were identified on April 14 2026 using the dbCAN3 server [33] and three complementary algorithms: HMMER: dbCAN, DIAMOND: CAZy, and HMMER: dbCAN-sub. The assignment of proteins to glycoside hydrolase families was subsequently verified manually.

Lists of experimentally characterized enzymes belonging to the GH5 and GH10 families were compiled using information available in the CAZy database [32]. The amino acid sequences of the GH5_5 and GH10 proteins encoded in the genome of strain T20PH1^T^ were used as queries to search for homologous proteins in the GenPept database (non-redundant protein sequences) using BLASTP. Database searches were performed between April and June 2026. For the GH5 family, a dataset was compiled using the 50 closest homologues of protein AC4GDJ_02660 identified by BLASTP together with 32 GH5_5 proteins analyzed previously [34]. The dataset was further supplemented with ten prokaryotic and ten most closely related eukaryotic representatives of the GH5_5 subfamily with experimentally verified cellulase activity [35]. For the GH10 family, datasets were constructed using the closest homologues of proteins AC4GDJ_00455, AC4GDJ_07495, AC4GDJ_08565, AC4GDJ_16910 and AC4GDJ_21055 identified by BLASTP.

Multiple sequence alignments were generated manually using BioEdit version 7.2 while taking into account pairwise sequence alignments obtained with BLASTP. After removal of highly variable regions, the resulting alignments were used for phylogenetic reconstruction with the PROTPARS algorithm implemented in the PHYLIP package [36]. Node support values were estimated from 100 bootstrap replicates generated using the programs SEQBOOT, PROTPARS and CONSENSE.

### 2.6. Phylogenomic Analysis

A genome-based tree was reconstructed using multiple sequence alignments of 120 single-copy marker genes, as implemented in GTDB-Tk v. 2.7.1 with the GTDB v. 232 reference database [37,38]. Average nucleotide identity (ANI) and average amino acid identity (AAI) were calculated using the Enveomics Gateway (Konstantinidis Lab, Paris), and the Genome-to-Genome Distance Calculator (GGDC) v. 3.0 was employed as an alternative digital DNA–DNA hybridization (dDDH) method for genome-to-genome comparisons [39].

## 3. Results and Discussion

### 3.1. Cell Morphology and Physiology

**Figure 1:**
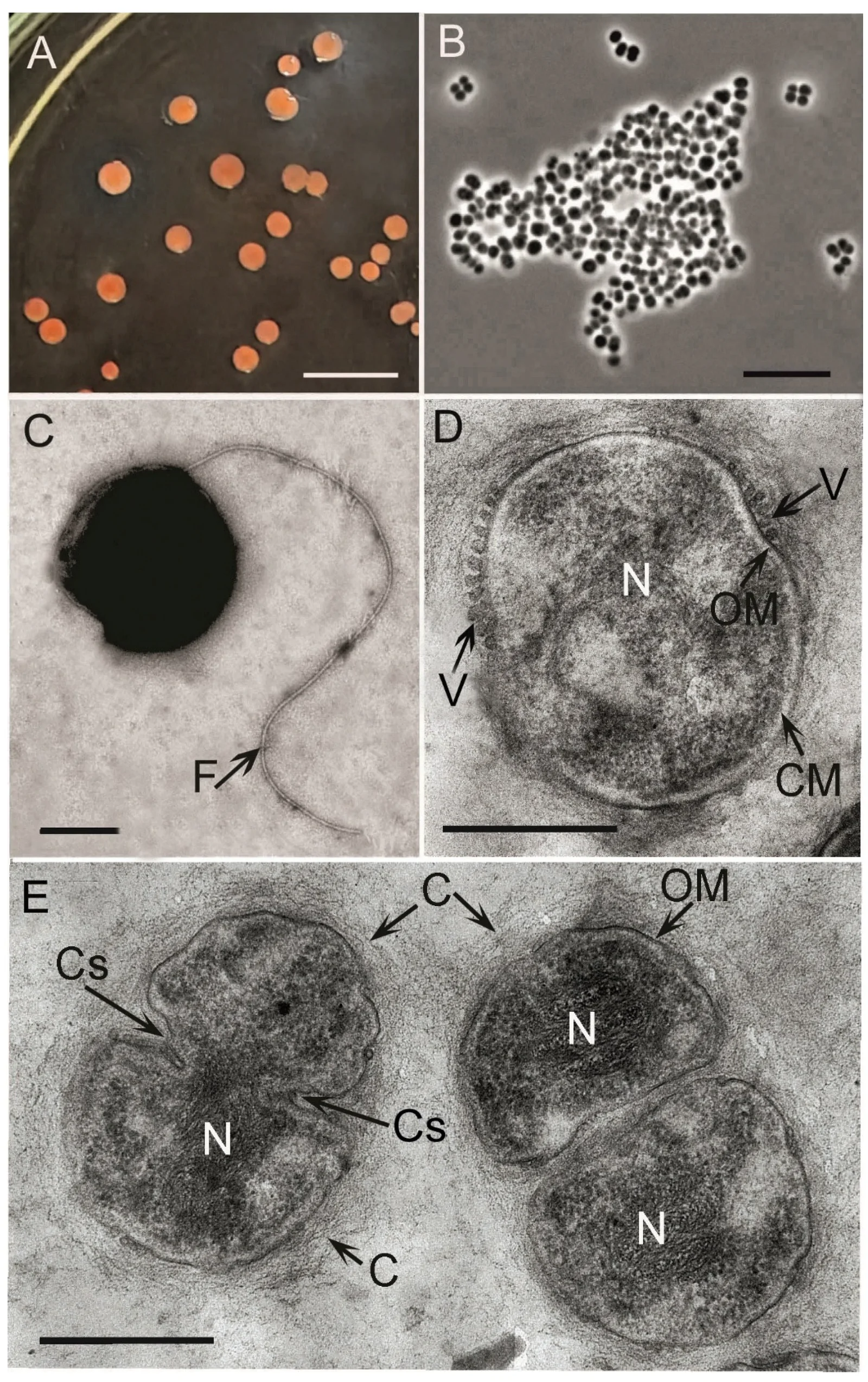
(**A**) Colonies produced by strain T20PH1^T^ on phytagel-solidified medium M31. (**B**) Phase-contrast micrograph of cells of strain T20PH1^T^ in 7-day-old culture. (**C**) Electron micrograph of a negatively stained cell showing a flagellum. (**D**,**E**) Electron micrographs of ultrathin cell sections of strain T20PH1^T^. Abbreviations: CM, cytoplasmic membrane; OM, outer membrane; N, nucleoid; F, flagellum; V, vesicles; Cs, constriction; C, capsule. Scale bars: (**A**) 1 cm, (**B**) 10 µm, (**C**–**E**) 0.5 µm.

On phytagel-solidified medium M31, strain T20PH1^T^ formed small (2–4 mm), circular, pink-to-red pigmented colonies (Figure 1A). The latter were composed of spherical cells, 0.9–1.8 μm in diameter, which occurred singly or were assembled in small and large aggregates (Figure 1B). Motile cells with one flagellum (Figure 1C) were observed in young (1–3 days-old) liquid cultures only. Cells reproduced by binary fission, were covered by capsules, and displayed a Gram-negative plan of cell organization (Figure 1D,E). Highly similar morphology and cell ultrastructure were reported for other described members of the order *Phycisphaerales*, i.e., *Tepidisphaera mucosa* 2842^T^, *Fontivita pretiosa* B-254^T^, and ‘*Humisphaera borealis*’ M1803^T^ [21,23,24]. A unique feature of cell organization in strain T20PH1^T^, however, was occurrence of small (~40–50 nm) equally spaced spherical vesicles, which were distributed over the cell surface (Figure 1D).

Growth occurred in the temperature range between 15 and 35 °C, with an optimum at 25–30 °C. The pH range for growth was pH 5.5–8.0, with an optimum at pH 6.0–7.0.

**Figure 2:**
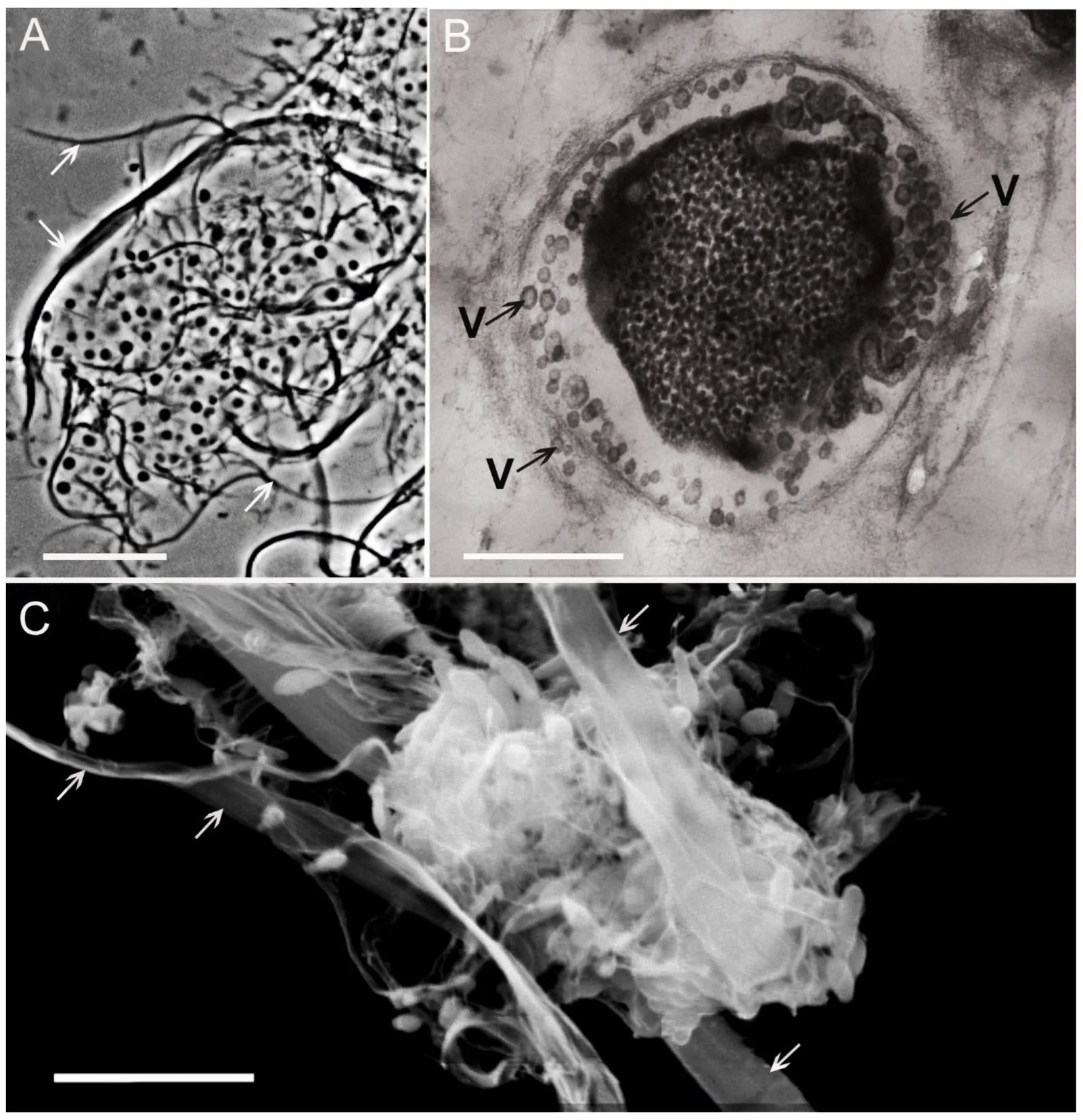
Observation of cells of strain T20PH1^T^ growing on fibrous cellulose used in cultivation experiments as a source of carbon. (**A**) Phase-contrast micrograph of cells attached to micro-fibrils of cellulose. (**B**) Ultrathin cell section of a cellulose-grown cell excreting multiple membrane vesicles. (**C**) Scanning electron micrograph of micro-colonies produced by strain T20PH1^T^ on micro-fibrils of cellulose. White arrows point to micro-fibrils of cellulose. Black arrows point to membrane vesicles (V). Scale bars: (**A**,**C**) 10 µm, (**B**) 0.5 µm.

Strain T20PH1^T^ was an aerobic chemoorganotroph capable of utilizing a relatively broad range of plant-derived carbohydrates. Growth was observed on arabinose, xylose, L-rhamnose, cellobiose and galactose. Among polymeric substrates, this planctomycete utilized xylan, xanthan gum, starch, lichenan and chitin. Notably, strain T20PH1^T^ was capable of growth on several forms of cellulose, including microcrystalline, carboxymethyl and fibrillar cellulose. Figure 2 (panels A and C) shows microscopic images of cells of strain T20PH1^T^ producing large aggregates that are tightly attached to micro-fibrils of cellulose. These cells excrete a large number of vesicles, which are nicely seen in ultrathin sections of cell grown on fibrous cellulose (Figure 2B).

In contrast, no growth was detected on trehalose, melibiose, raffinose, sucrose, ribose, melezitose, fructose, sorbose or maltose. The strain was also unable to utilize fucoidan, pullulan, laminarin, chondroitin sulfate or pectin. Among the organic acids tested, only pyruvate and fumarate supported growth, whereas acetate, formate, propionate, butyrate, lactate and citrate were not utilized as sole carbon sources.

In API 20NE tests, strain T20PH1^T^ reduced nitrate to nitrite and showed positive reactions for glucose fermentation, β-galactosidase activity, esculin hydrolysis and gelatin hydrolysis. This bacterium was oxidase-positive and catalase-negative.

Regarding antibiotic susceptibility, strain T20PH1^T^ was resistant to neomycin, streptomycin, gentamicin, lincomycin, kanamycin and rifampicin. The strain was sensitive to chloramphenicol and ampicillin, and showed weak sensitivity to tetracycline.

### 3.2. 16S rRNA Gene-Based Identification

The comparative 16S rRNA gene sequence analysis placed strain T20PH1^T^ within the family *Tepidisphaeraceae* of the order *Tepidisphaerales*. Currently, only three representatives of this order and family have been described. The closest relative for strain T20PH1^T^ is the thermophilic planctomycete *Fontivita pretiosa* B-254^T^ [24], with 88.9% 16S rRNA sequence identity. Another moderate thermophile, *Tepidisphaera mucosa* 2842^T^ [21], shared 88.36% similarity, while ‘*Humisphaera borealis*’ M1803^T^ [23], isolated from a peatbog humic lake, showed 88.59% similarity.

### 3.3. Genome Characteristics

**Figure 3:**
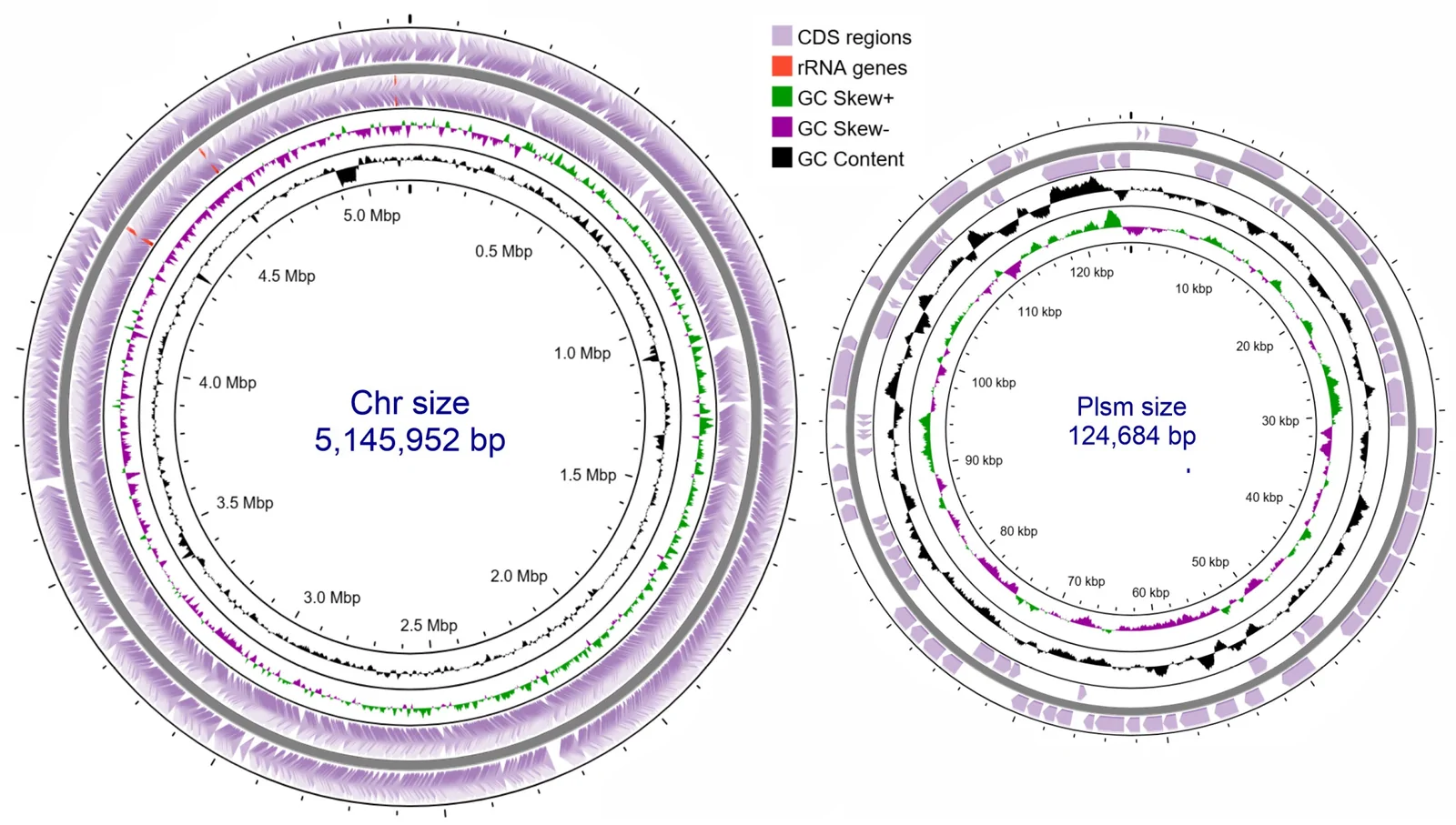
Circular map of the chromosome (chr) and plasmid (plsm) sequences of strain T20PH1^T^. The innermost circle indicates the genome coordinates in base pairs. The two inner rings show the GC skew and GC content, respectively. The outer rings display the distribution of CDS and rRNA genes on the forward and reverse strands.

The genome of strain T20PH1^T^ consisted of a 5,145,952 bp chromosome and a circular plasmid of 124,684 bp. The G + C content of the chromosomal DNA was 65.97 mol% and the corresponding value for the plasmid was 66.75 mol% (Figure 3). A total of 4415 potential protein-coding genes and 64 tRNA genes were predicted. The genome contained two rRNA operons. One displayed the canonical gene order (16S–23S–5S). The second operon was “unlinked” with the 16S rRNA gene located approximately 510 kb apart from a 23S-5S rRNA gene pair. The linked and unlinked 16S rRNA gene copies in strain T20PH1^T^ were identical. Within the order *Tepidisphaerales*, *T. mucosa* 2842^T^ and *F. pretiosa* B-254^T^ each contained a single unlinked rRNA operon, while ‘*H. borealis*’ showed a pattern similar to strain T20PH1^T^, with one canonical and one unlinked operon. Unlinked rRNA operons have been documented in various planctomycetes from different families [40,41,42,43,44,45].

### 3.4. Phylogenomic Analysis and Environmental Distribution

**Figure 4:**
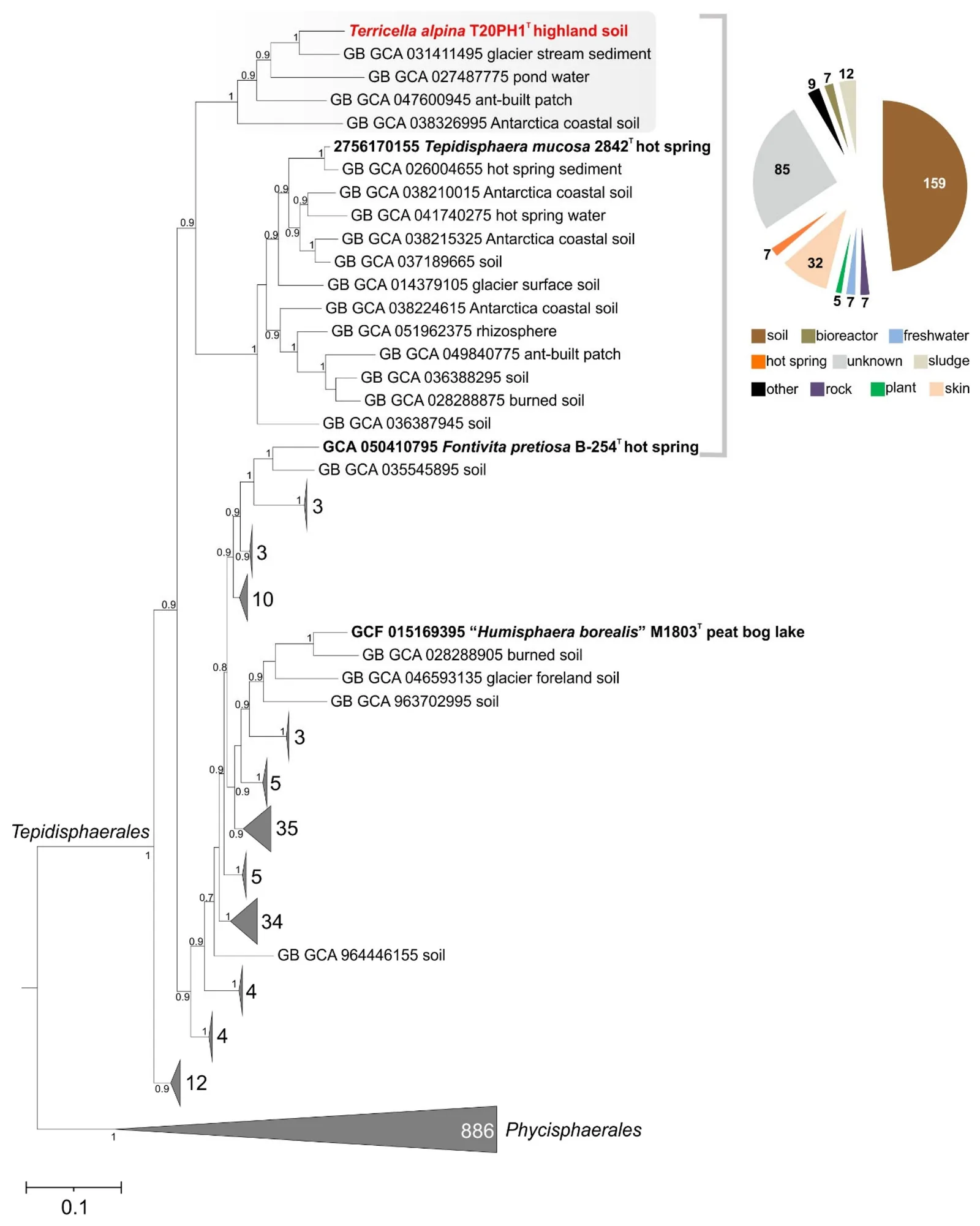
Phylogenomic tree constructed based on a concatenated alignment of 120 conserved marker proteins from strain T20PH1^T^, *Tepidisphaera mucosa* 2842^T^, *Fontivita pretiosa* B-254^T^, ‘*Humisphaera borealis*’ M1803^T^, and other representatives of the order *Tepidisphaerales* available in GTDB v.232. All cultivated representatives are shown in bold. The strain described in this study is highlighted in red, and its corresponding genus-level clade is indicated by a grey box. All 54 genomes of anammox planctomycetes belonging to the family *Scalinduaceae* available in GTDB v.232 were used as an outgroup. Bootstrap values > 70% are indicated at the nodes. Scale bar represents 0.1 amino acid substitutions per site. The accompanying pie chart shows the environmental distribution of all 16S rRNA gene sequences assigned to the family *Tepidisphaeraceae* in the SILVA database v.138.2, corresponding to the clade highlighted in brackets in the GTDB tree.

Based on the phylogenomic analysis, strain T20PH1^T^ was most closely related to *T. mucosa* 2842^T^ (Figure 4). Following the proposed minimal standards for the use of genome data for the taxonomy of prokaryotes, we determined the overall genome similarity, ANI and AAI values between strain T20PH1^T^ and other members of the family *Tepidisphaeraceae*. Strain T20PH1^T^ shared 21.1 ± 2.4% overall genome similarity with *T. mucosa* 2842^T^, while the ANI value calculated for the genomes of these planctomycetes was 78%. The GGDC values for *F. pretiosa* and ‘*H. borealis*’ were 18.2 ± 2.4% and 20.2 ± 2.4%, respectively, with corresponding ANI values of 75% and 76%. The AAI between the T20PH1^T^ genome and those of *T. mucosa* 2842^T^, *F. pretiosa* B-254^T^, and ‘*Humisphaera borealis*’ M1803^T^ was 52.6%, 51.7%, and 49.9%, respectively. While GGDC and ANI are used for species delineation (thresholds of 70% and 95–96%, respectively), all pairwise values for strain T20PH1^T^ were far below these thresholds. For genus delineation, AAI (threshold 60–65%) and the tree topology (Figure 4) confirm that strain T20PH1^T^ represents a novel genus and species within the family *Tepidisphaeraceae* [46,47].

To gain insights into the environmental distribution of *Tepidisphaerales* members, we analyzed both genome-resolved and 16S rRNA gene datasets. In the current GTDB (release 232), the order *Tepidisphaerales* was represented by 169 high-quality genomes and MAGs. Based on the phylogenomic tree topology, strain T20PH1^T^ formed a distinct lineage together with *Tepidisphaera mucosa* 2842^T^ (Figure 4). This clade comprised 18 genomes in GTDB originating from diverse environments, including terrestrial habitats [48,49,50], hot spring waters and sediments [51,52], freshwaters [53], Antarctic and glacier-associated environments [54,55], and ant-constructed soil patches [56]. Because the genomic dataset was limited, we further examined the distribution of related 16S rRNA gene sequences in the SILVA database (release 138.2), which contained 6378 sequences assigned to the order *Tepidisphaerales*. It should be noted that the taxonomic structure of SILVA differed from that of GTDB. In particular, representatives of ‘*H. borealis*’ and *F. pretiosa* are assigned to the WD2101 group rather than to *Tepidisphaeraceae*. Previous analyses of SILVA sequences affiliated with this group showed that the majority originated from terrestrial environments, particularly soils [23,24]. In the present study, we focused specifically on the SILVA clade named *Tepidisphaeraceae* corresponding to the T20PH1^T^ and *Tepidisphaera* lineage of the GTDB tree (Figure 4). This clade comprised 328 16S rRNA gene sequences. As shown in Figure 4, soil-derived records accounted for the largest fraction (48%), whereas environmental metadata were unavailable for 25% of the dataset. The second most abundant category (9.5%) included sequences recovered from skin samples of children with atopic dermatitis and experimental mice [57,58]. Additional records originated from sludge, freshwater habitats, rocks, plants, hot springs and bioreactors. Despite the apparent prevalence of this lineage in various soils, strain T20PH1^T^ was the first cultivated isolate from this ecosystem.

### 3.5. Genome-Encoded Metabolism

The genome encoded all major metabolic pathways typical for aerobic chemoorganotrophic bacteria, including glycolysis, the tricarboxylic acid cycle, the pentose phosphate pathway, and oxidative phosphorylation. Genes required for biosynthesis of all amino acids were identified. The genome contained a complete peptidoglycan biosynthesis pathway, including *murA*, *murB*, *murC*, *murD*, *murE*, *murF*, *mraY*, and *murG*, together with penicillin-binding proteins, including *ftsI*. Genes involved in lipopolysaccharide biosynthesis and amino sugar metabolism were also present, indicating a fully developed Gram-negative-like cell envelope. A broad repertoire of ABC transporters was identified (53 transporter genes based on KEGG), including systems predicted to transport amino acids, peptides, and carbohydrates. The genome also encoded multiple predicted glycosidases, proteases, and esterases involved in degradation of complex extracellular substrates.

Genes involved in chemotaxis and flagellar assembly were present, including *cheA*, *cheB*, *cheR*, *cheW*, and *cheY*, together with multiple methyl-accepting chemotaxis proteins. The flagellar apparatus was represented by genes encoding components of the basal body and motor complex, including *fliF*, *fliG*, *fliM*, *fliN*, *flhA*, *flhB*, *motA*, and *motB*, as well as multiple *flg* genes associated with rod and hook assembly. These findings were consistent with the observed motility of the strain.

A complete C40 carotenoid biosynthesis pathway and terpenoid backbone biosynthesis pathway were identified, suggesting the capacity for carotenoid pigment production and isoprenoid metabolism, consistent with the pink pigmentation of the strain.

Notably, no bacterial microcompartment (BMC) loci were detected in the genome of strain T20PH1^T^. This finding is unexpected because BMC-associated PVM clusters appear to be conserved among the currently characterized members of the order *Tepidisphaerales*. In our previous study, the presence of metabolosome-like BMCs and a corresponding PVM gene cluster was demonstrated in ‘*Humisphaera borealis*’ M1803^T^ [23]. In addition, re-analysis of the available genomes of *Tepidisphaera mucosa* and *Fontivita pretiosa* performed in the present study using BMC Caller and BLAST-based searches revealed the presence of PVM-type BMC loci in both organisms. In contrast, no comparable PVM cluster or canonical BMC shell protein genes could be detected in strain T20PH1^T^. This observation is particularly intriguing because strain T20PH1^T^ was capable of growth on L-rhamnose and possessed genes required for L-rhamnose utilization, including α-L-rhamnosidase, L-rhamnose isomerase, and L-rhamnulokinase. Furthermore, genes encoding aldehyde dehydrogenase and L-lactate dehydrogenases, which may participate in the conversion of lactaldehyde-derived intermediates, were present in the genome. These findings suggest that L-rhamnose catabolism in strain T20PH1^T^ proceeds without a canonical PVM-type microcompartment and instead relies on cytosolic enzymatic processing of metabolic intermediates.

Using antiSMASH, nine putative secondary metabolite biosynthetic gene clusters were identified in the genome of strain T20PH1^T^: one RRE-containing, one NRPS-like, three terpene precursors, two arylpolyene, one cytokinin-related, and one oligosaccharide-associated cluster. They were distributed across the chromosome. Among planctomycetes, detectable similarity was found only for one arylpolyene cluster homologous to a region in *Algisphaera agarilytica* DSM 103725. Arylpolyene clusters, besides carotenoids, typically protect against oxidative stress via pigment production. Terpene clusters relate to isoprenoid-derived metabolites involved in membrane structure or ecological interactions. NRPS-like and RRE-containing clusters suggest a limited capacity for peptide-based secondary metabolism. The cytokinin and oligosaccharide clusters may be involved in signaling and carbohydrate-related surface modification, respectively.

### 3.6. Genomic Determinants of Cellulolytic and Xylanolytic Capabilities

Putative glycoside hydrolases encoded in the genome of strain T20PH1^T^ were identified using the dbCAN3 meta-server. Manual inspection of the annotated proteome indicated that members of the GH5 and GH10 families were the most likely candidates for cellulase and xylanase activities, respectively.

Strain T20PH1^T^ encoded three GH5 family proteins assigned to two subfamilies: GH5_5 (locus tag AC4GDJ_02660) and GH5_47 (AC4GDJ_00905 and AC4GDJ_07785). To date, the GH5_47 subfamily comprises only a single biochemically characterized member, a β-1,3-1,6-endoglucanase (EC 3.2.1.39 and EC 3.2.1.75) from *Saccharophagus degradans* 2–40 [35]. Consequently, prediction of the enzymatic functions of AC4GDJ_00905 and AC4GDJ_07785 remains difficult. In contrast, the GH5_5 subfamily includes 76 experimentally characterized proteins, the vast majority of which exhibit cellulase activity (EC 3.2.1.4), including all ten reported prokaryotic representatives. Therefore, AC4GDJ_02660 was considered the most likely cellulase candidate and was selected for further phylogenetic analysis.

**Figure 5:**
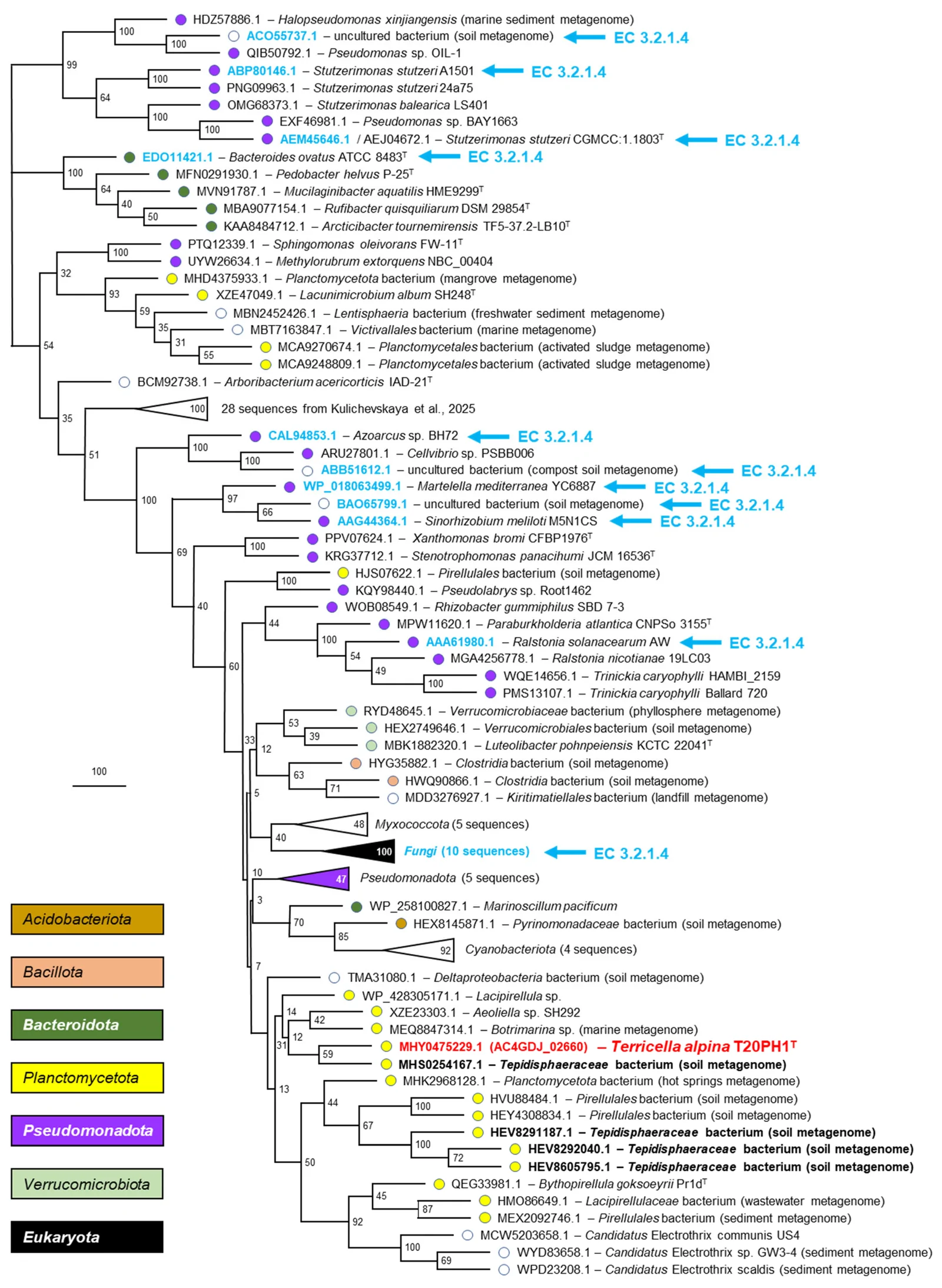
Maximum parsimony phylogenetic tree of glycoside hydrolase subfamily GH5_5. Statistical support for nodes was assessed by bootstrap analysis, with the number of supporting pseudoreplicates (out of 100) shown at each node. Triangles indicate protein clusters. Bootstrap support values for each cluster are shown within the corresponding triangle, and the number of proteins in each cluster is indicated nearby. Phylogenetic affiliations of proteins are color-coded. The positions of experimentally characterized cellulases (EC 3.2.1.4) are indicated by blue arrows. The tree includes 116 proteins. The cluster containing 28 sequences has been collapsed for clarity; its detailed internal topology was previously reported by Kulichevskaya et al., 2025 [34].

In the phylogenetic tree, AC4GDJ_02660 grouped within a strongly supported cluster (100% bootstrap support) containing numerous homologues, including 16 experimentally characterized cellulases from bacteria and fungi (Figure 5). Its closest homologue (MHS0254167.1) originated from a soil-derived metagenome-assembled genome assigned to the *Tepidisphaeraceae* family. Three additional homologues from soil-derived *Tepidisphaeraceae* MAGs (HEV8605795.1, HEV8292040.1 and HEV8291187.1; BioProject PRJNA983538) formed a well-supported subcluster within the same lineage. These findings suggest that GH5_5 cellulases may be widespread among members of the *Tepidisphaeraceae* family.

Strain T20PH1^T^ encoded eight GH10 family proteins. One of them (locus tag AC4GDJ_14160) belonged to a highly divergent subfamily characterized by the absence of both catalytically essential glutamate residues [59]. The remaining seven proteins were considered potential xylanase candidates. Five of them (AC4GDJ_00455, AC4GDJ_07495, AC4GDJ_08565, AC4GDJ_16910 and AC4GDJ_21055) formed a coherent subfamily and were therefore selected for phylogenetic analysis. This subfamily contains only a single biochemically characterized representative, a β-xylanase (EC 3.2.1.8) with broad substrate specificity recovered from an uncultured bacterium inhabiting marine hydrothermal sediments [60]. The remaining two GH10 proteins of strain T20PH1^T^ (AC4GDJ_00405 and AC4GDJ_00470) showed highest similarity to experimentally characterized β-xylanases from *Bacillus pumilus* (AAM21605.1; 29% amino acid sequence identity) and *Melioribacter roseus* (AFN75725.1; 47%), respectively.

**Figure 6:**
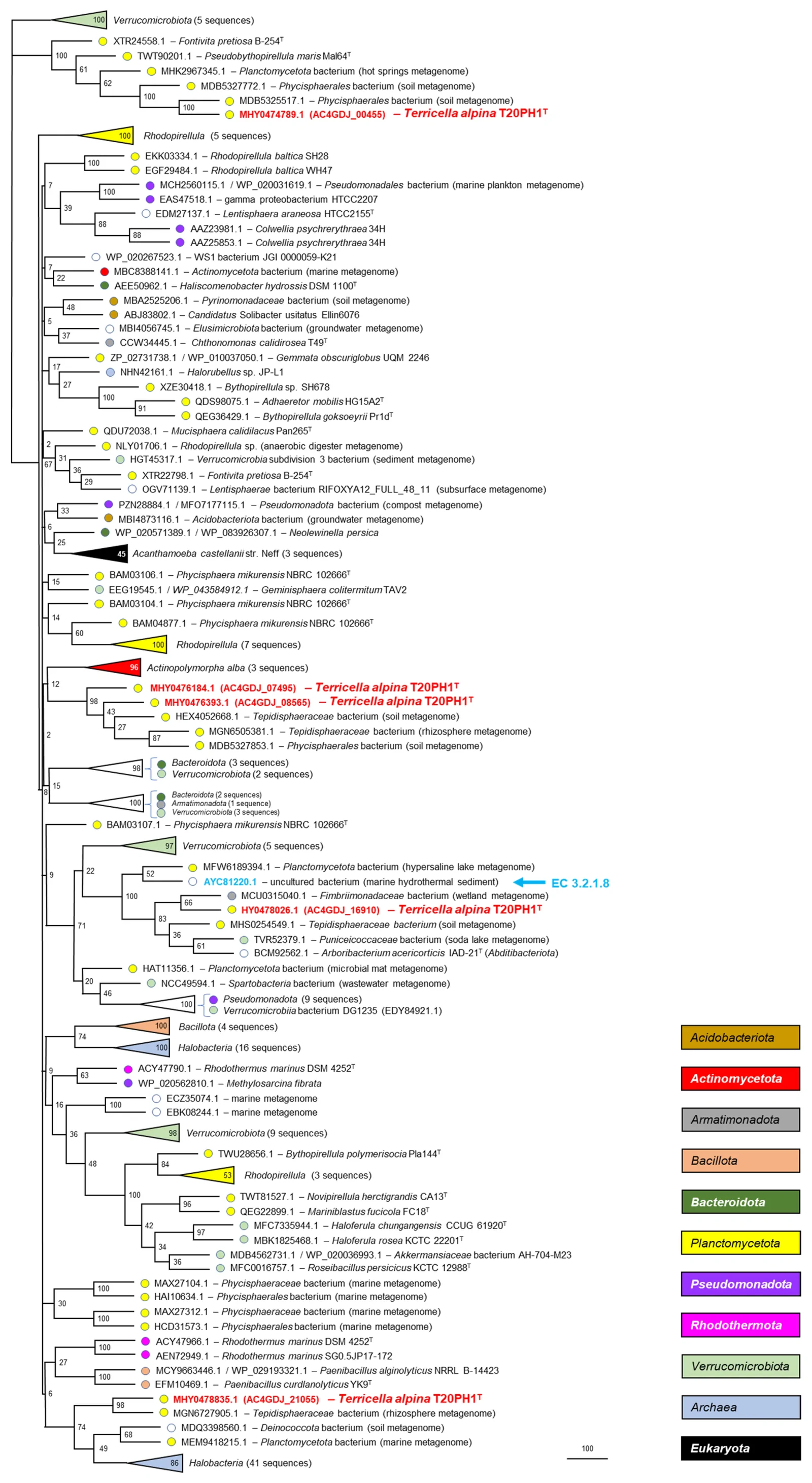
Maximum parsimony phylogenetic tree of glycoside hydrolase family GH10. Statistical support for nodes was assessed by bootstrap analysis, with the number of supporting pseudoreplicates (out of 100) shown at each node. Triangles indicate protein clusters. Bootstrap support values for each cluster are shown within the corresponding triangle, and the number of proteins in each cluster is indicated nearby. Phylogenetic affiliations of proteins are color-coded. The position of the experimentally characterized β-xylanase (EC 3.2.1.8) with broad specificity is indicated by a blue arrow. The tree includes 197 proteins.

The inferred phylogenetic tree indicated that the GH10-encoding genes of strain T20PH1^T^ had distinct evolutionary origins (Figure 6). The only apparent exception was the AC4GDJ_07495/AC4GDJ_08565 pair, which likely resulted from a recent gene duplication event. Notably, the genome of *Fontivita pretiosa* B-254^T^ contains five GH10-encoding genes [35]. Collectively, these observations suggest that the GH10 repertoire of *Tepidisphaeraceae* members has been shaped by multiple gene acquisition and diversification events. The presence of numerous GH10 genes in both strain T20PH1^T^ and *F. pretiosa* B-254^T^ points to an important role of xylan degradation in the ecology of these organisms.

In summary, phenotypic and genomic characteristics of strain T20PH1^T^ were clearly distinct from those of the three earlier described *Tepidisphaeraceae* members (Table 1). Based on this distinction, we propose to classify this soil-inhabiting planctomycete as representing a novel genus and species, *Terricella alpina* gen. nov., sp. nov. The descriptions of the novel genus and species are given below.

**Description of *Terricella* gen. nov.**

*Terricella* (Ter.ri.sphae’ra. L. fem. n. *terra* soil, earth; L. fem. n. *sphaera* a sphere; N.L. fem. n. *Terricella*, a spherical bacterium inhabiting soil).

Gram-stain-negative, spherical cells reproducing by binary fission. Cells occur singly, in pairs or in small aggregates. Motile. Pink-pigmented. Aerobic. Oxidase-positive and catalase-negative. Mesophilic and neutrophilic. Chemoorganotrophic. Sugars, polysaccharides and cellulose are utilized as growth substrates. The genus belongs to the family *Tepidisphaeraceae* of the order *Tepidisphaerales*. The type species is *Terricella alpina*.

**Description of *Terricella alpina* sp. nov.**

*Terricella alpina* (al.pi’na. L. fem. adj. *alpina*, pertaining to alpine environments).

Exhibits the following properties in addition to those given in the genus description. Cells are 0.9–1.8 μm in diameter. Growth occurs at 15–35 °C (optimum 25–30 °C) and at pH 5.5–8.0 (optimum pH 6.0–7.0). Growth is observed on arabinose, xylose, L-rhamnose, cellobiose and galactose. Utilizes xylan, xanthan gum, starch, lichenan, chitin, microcrystalline cellulose, fibrous cellulose and carboxymethyl cellulose. Pyruvate and fumarate are utilized as growth substrates. No growth occurs on trehalose, melibiose, raffinose, sucrose, ribose, melezitose, fructose, sorbose, maltose, acetate, formate, propionate, butyrate, lactate, citrate, pectin, fucoidan, pullulan, chondroitin sulfate or laminarin.

Positive for nitrate reduction, glucose fermentation, β-galactosidase activity, esculin hydrolysis and gelatin hydrolysis (API 20NE). Oxidase-positive and catalase-negative. Resistant to neomycin, streptomycin, gentamicin, lincomycin, kanamycin and rifampicin; sensitive to ampicillin and chloramphenicol; weakly sensitive to tetracycline.

The DNA G + C content of the type strain calculated from the genome sequence is 65.97 mol%. The type strain, T20PH1^T^ (=LMG 34157^T^ = KCTC 102499^T^), was isolated from alpine meadow soil collected at an altitude of 1800 m in the North Caucasus Mountains, Republic of North Ossetia-Alania, Russia.

## 4. Conclusions

In summary, this study expands our current knowledge of the phenotypic and metabolic diversity within the planctomycetal order *Tepidisphaerales*. Strain T20PH1^T^ represents a novel genus and species within the family *Tepidisphaeraceae*, for which the name *Terricella alpina* gen. nov., sp. nov. is proposed. It is the first cultivated soil-derived representative of the class *Phycisphaerae*, a group widely detected in soils but long elusive in cultivation. The ability of *T. alpina* T20PH1^T^ to degrade cellulose, xylan and other plant-derived polysaccharides suggests a potential contribution of this lineage to plant-derived organic matter turnover in soils. The genomic and physiological characterization of this isolate provides a basis for future investigations of its unique biological features, including the intriguing extracellular vesicle structures that densely surround the cells.

## Figures and Tables

**Table 1:** Major characteristics that distinguish strain T20PH1^T^, *Fontivita pretiosa* B-254^T^ [24], *Tepidisphaera mucosa* 2842^T^ [21], ‘*Humisphaera borealis*’ M1803^T^ [23].

Characteristics	T20PH1^T^	*F. pretiosa* B-254^T^	*T. mucosa* 2842^T^	‘*H. borealis*’ M1803^T^
Isolation source	alpine soil, North Osetia region, Russia	Hot spring, Goryachinsk, Baikal region, Russia	Hot spring, Goryachinsk, Baikal region, Russia	Boreal eutrophic lake, Vologda region, Russia
Cell size	0.9–1.8	0.6–1	0.5–1.2	1–3
Color (conditions)	pink (aero)	creamy or beige (aero)/creamy or white (anaero)	pink (aero)/creamy or white (anaero)	pink (aero)
min/opt/max,T	15/25–30/35	30/50–54/57	20/47–50/56	10/20–25/30
min/opt/maxpH	5.5/6.0–7.0/8.0	5.1/6.6–7.1/8.4	4.5/7.0–7.5/8.5	5.5/6.5–7.0/8.0
Catalase	–	–	–	+
Oxidase	+	+	+	+
Relation to oxygen	Aerobe	Facultative anaerobe	Facultative anaerobe	Microaerobe and aerobe
**Growth on substrates**				
Sucrose	–	+	+	–
Fructose	–	+	–	–
Arabinose	+	+	–	nd
Trehalose	–	+	+	–
Pectin	–	–	+	+
Cellulose	+	–	nd	–
Xylan	+	+	–	+
**Products of fermentation**	–	Ethanol, acetate, H2 and CO2	Acetate and propionate	–
**G + C, mol%**	65.9	64	53.5	63.1
**Genome size, Mb**	5.27	5.54	3.43	7.19

## Data Availability

The annotated chromosome and plasmid sequences of strain T20PH1^T^ have been deposited in GenBank under the accession number JBZEMQ000000000.
