# Correction: Ivanova et al. *Terricella alpina* gen. nov., sp. nov., a Cellulolytic Planctomycete Representing the First Described Soil-Inhabiting Member of the Class *Phycisphaerae*. *Life* 2026, *16*, 1222

**DOI:** 10.3390/life16091534

**Published:** 2026-09-15

**Authors:** Anastasia A. Ivanova, Irina S. Kulichevskaya, Daniil G. Naumoff, Gennady S. Kachmazov, Natalia E. Suzina, Svetlana N. Dedysh

**Affiliations:** 1 Winogradsky Institute of Microbiology, Research Center of Biotechnology of the Russian Academy of Sciences, Moscow 119071, Russia; kulich2@mail.ru (I.S.K.); daniil_naumoff@yahoo.com (D.G.N.); dedysh@mail.ru (S.N.D.); 2 State Budgetary Educational Institution, Republican Physics and Mathematics Lyceum-Boarding School Named After M.K. Abaev, Republic of North Ossetia—Alania, Vladikavkaz 362021, Russia; kgssogutfi@yandex.ru; 3 G.K. Skryabin Institute of Biochemistry and Physiology of Microorganisms, Pushchino Scientific Center for Biological Research of the Russian Academy of Sciences, Pushchino 142290, Russia; suzina_nataliya@rambler.ru

**Text Correction**

There was an error in the original publication [1]. The authors regret that after publication it was brought to our attention that the generic name *Terrisphaera* proposed in this article had already been validly published for another bacterial genus. According to the International Code of Nomenclature of Prokaryotes, this generic name cannot be used for the taxon described in our paper. Therefore, the replacement name *Terricella* gen. nov. is proposed. The species epithet *alpina* remains unchanged, and the correct name is *Terricella alpina* gen. nov., sp. nov.

A correction has been made to the **Title, Abstract, Keywords, Taxon description and Conclusions**. The authors state that the scientific conclusions are unaffected. This correction was approved by the Academic Editor. The original publication has also been updated.

Corrected name of the newly proposed genus—*Terricella* gen. nov.

*Terricella* (Ter.ri.cel’la. L. fem. n. *terra*, soil, earth; L. fem. n. *cella*, a cell; N.L. fem. n. *Terricella*, a cell from soil).

The type species is *Terricella alpina*.

Corrected name of the newly proposed species—*Terricella alpina* sp. nov.

*Terricella alpina* (al.pi’na. L. fem. adj. *alpina*, pertaining to alpine environments).

The type strain is strain T20PH1^T^ (=LMG 34157^T^ = KCTC 102499^T^).

**Figure Corrections**

In the figures below, the organism name has also been changed from *Terrisphaera alpina* to *Terricella alpina*, while the figure captions remain unchanged.

**Figure 4:**
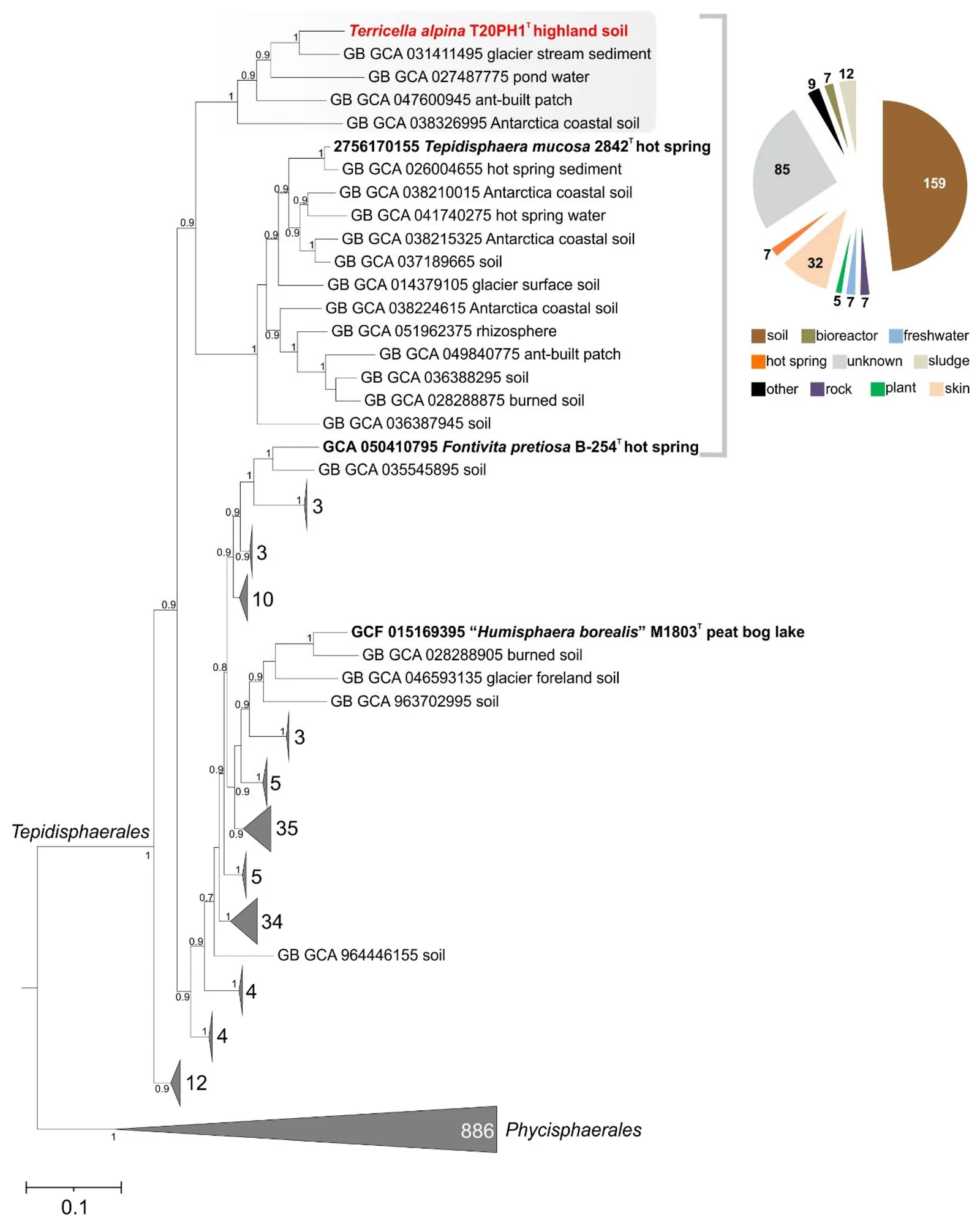
Phylogenomic tree constructed based on a concatenated alignment of 120 conserved marker proteins from strain T20PH1^T^, *Tepidisphaera mucosa* 2842^T^, *Fontivita pretiosa* B-254^T^, ‘*Humisphaera borealis*’ M1803^T^, and other representatives of the order *Tepidisphaerales* available in GTDB v.232. All cultivated representatives are shown in bold. The strain described in this study is highlighted in red, and its corresponding genus-level clade is indicated by a grey box. All 54 genomes of anammox planctomycetes belonging to the family *Scalinduaceae* available in GTDB v.232 were used as an outgroup. Bootstrap values > 70% are indicated at the nodes. Scale bar represents 0.1 amino acid substitutions per site. The accompanying pie chart shows the environmental distribution of all 16S rRNA gene sequences assigned to the family *Tepidisphaeraceae* in the SILVA database v.138.2, corresponding to the clade highlighted in brackets in the GTDB tree.

**Figure 5:**
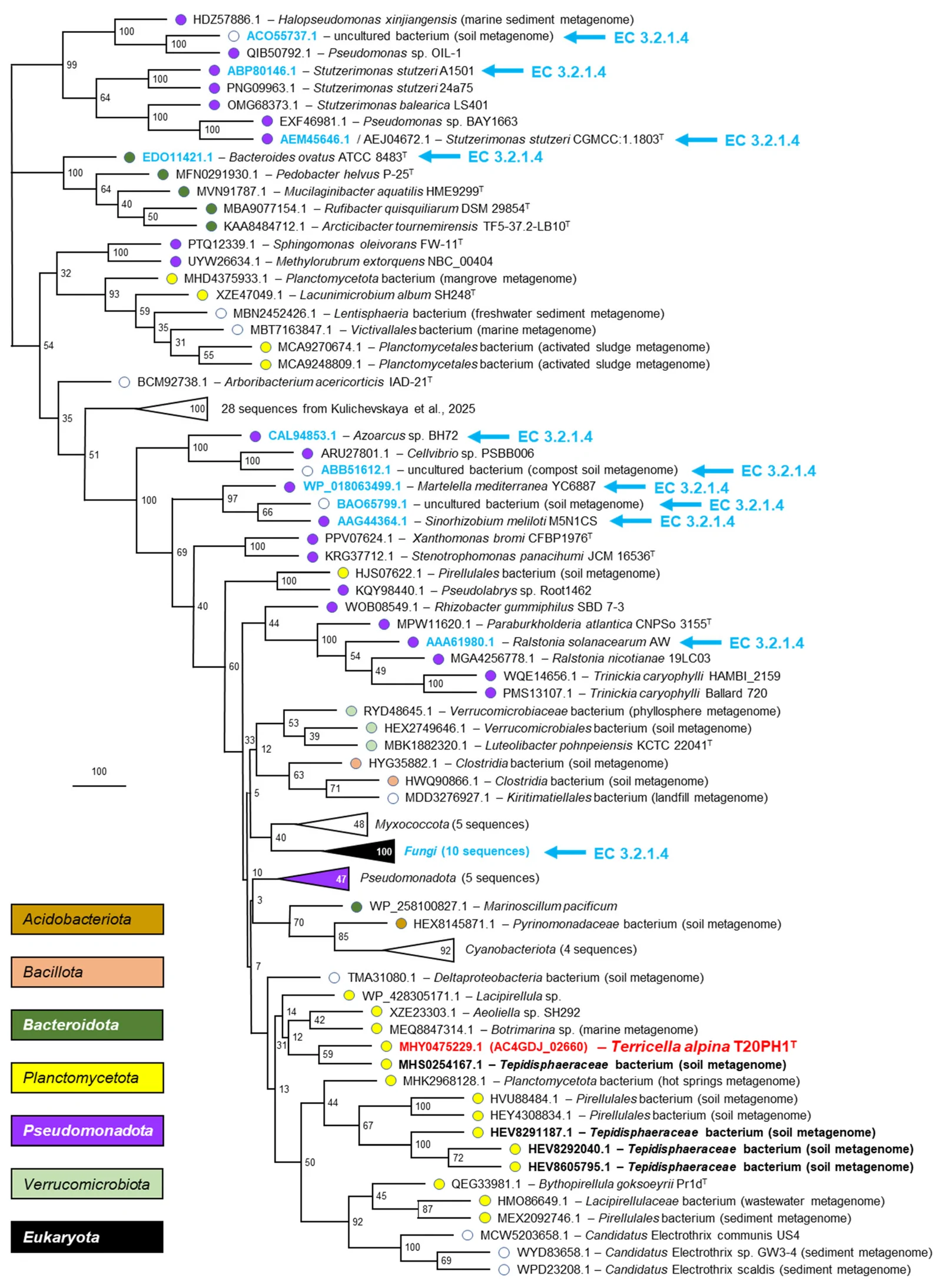
Maximum parsimony phylogenetic tree of glycoside hydrolase subfamily GH5_5. Statistical support for nodes was assessed by bootstrap analysis, with the number of supporting pseudoreplicates (out of 100) shown at each node. Triangles indicate protein clusters. Bootstrap support values for each cluster are shown within the corresponding triangle, and the number of proteins in each cluster is indicated nearby. Phylogenetic affiliations of proteins are color-coded. The positions of experimentally characterized cellulases (EC 3.2.1.4) are indicated by blue arrows. The tree includes 116 proteins. The cluster containing 28 sequences has been collapsed for clarity; its detailed internal topology was previously reported by Kulichevskaya et al., 2025 [34].

**Figure 6:**
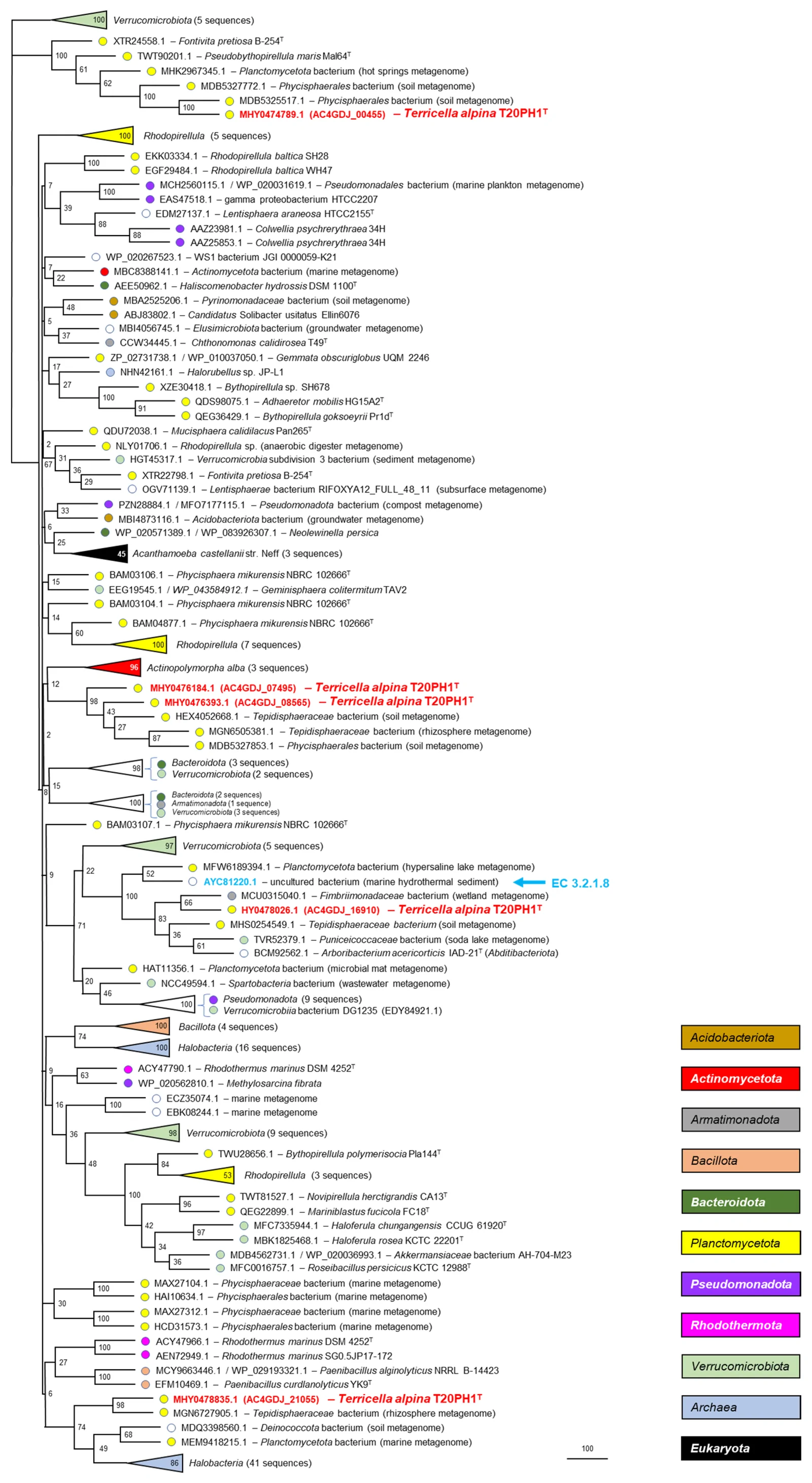
Maximum parsimony phylogenetic tree of glycoside hydrolase family GH10. Statistical support for nodes was assessed by bootstrap analysis, with the number of supporting pseudoreplicates (out of 100) shown at each node. Triangles indicate protein clusters. Bootstrap support values for each cluster are shown within the corresponding triangle, and the number of proteins in each cluster is indicated nearby. Phylogenetic affiliations of proteins are color-coded. The position of the experimentally characterized β-xylanase (EC 3.2.1.8) with broad specificity is indicated by a blue arrow. The tree includes 197 proteins.
